# Ectodysplasin A Pathway Contributes to Human and Murine Skin Repair

**DOI:** 10.1016/j.jid.2015.09.002

**Published:** 2016-05

**Authors:** Clare L. Garcin, Kenneth M. Huttner, Neil Kirby, Pascal Schneider, Matthew J. Hardman

**Affiliations:** 1 The Healing Foundation Centre, Faculty of Life Sciences, University of Manchester, Manchester, United Kingdom; 2 Edimer Pharmaceuticals Inc, Cambridge, Massachusetts, USA; 3 Department of Biochemistry, University of Lausanne, Lausanne, Switzerland

**Keywords:** EDA, ectodysplasin A, EDAR, ectodysplasin A receptor, HED, hypohidrotic ectodermal dysplasia, HC, hair cycle, HF, hair follicle, IFE, interfollicular epidermis, OVX, ovariectomized, WT, wild type, XLHED, X-linked hypohidrotic ectodermal dysplasia

## Abstract

The highly conserved ectodysplasin A (EDA)/EDA receptor signaling pathway is critical during development for the formation of skin appendages. Mutations in genes encoding components of the EDA pathway disrupt normal appendage development, leading to the human disorder hypohidrotic ectodermal dysplasia. Spontaneous mutations in the murine *Eda* (*Tabby*) phenocopy human X-linked hypohidrotic ectodermal dysplasia. Little is known about the role of EDA signaling in adult skin homeostasis or repair. Because wound healing largely mimics the morphogenic events that occur during development, we propose a role for EDA signaling in adult wound repair. Here we report a pronounced delay in healing in *Tabby* mice, demonstrating a functional role for EDA signaling in adult skin. Moreover, pharmacological activation of the EDA pathway in both *Tabby* and wild-type mice significantly accelerates healing, influencing multiple processes including re-epithelialization and granulation tissue matrix deposition. Finally, we show that the healing promoting effects of EDA receptor activation are conserved in human skin repair. Thus, targeted manipulation of the EDA/EDA receptor pathway has clear therapeutic potential for the future treatment of human pathological wound healing.

## Introduction

Hypohidrotic ectodermal dysplasia (HED) is a well-characterized human disease affecting the morphology and number of skin appendages, principally the hair follicles (HFs), teeth, and exocrine glands (Mikkola, 2009, Mikkola and Thesleff, 2003, Naito et al., 2002). HED, the most common of the ectodermal dysplasias, is caused by mutations to the ectodysplasin signaling pathway, which is essential for in utero development of ectoderm-derived appendages. The main axis of the pathway comprises the ligand ectodysplasin A (EDA, encoded in mice by *Tabby*), ectodysplasin A receptor (EDAR, encoded by *downless/Sleek*), and the adaptor molecule EDAR-associated protein with a death domain (encoded by *crinkled*). Mutations in any of these pathway components leads to human HED, which is phenocopied in mice (Cui and Schlessinger, 2006). Mutations in the X-linked *EDA* gene underlie most ectoderm dysplasia cases (X-linked HED [XLHED]) (Huttner, 2014, Monreal et al., 1998).

EDA, a member of the tumor necrosis factor family of signaling molecules, exists in two highly homologous isoforms, EDA1 and EDA2 (Bayes et al., 1998, Elomaa et al., 2001, Mikkola and Thesleff, 2003). EDA1 is specific for the type I transmembrane protein EDAR, whereas EDA2 is specific for the type III, X-linked transmembrane receptor (Mikkola and Thesleff, 2003, Yan et al., 2000). Mutations to EDA2 do not result in XLHED; however, this ligand is thought to play a role in hair loss during adulthood (Brosh et al., 2010). To invoke EDAR signaling, EDA ligands are shed from the cell surface before receptor binding. Receptor activation initiates association with the C-terminal death domain of EDAR-associated protein with a death domain, which creates a complex capable of interacting with tumor necrosis factor receptor-associated factors (Headon et al., 2001, Yan et al., 2000). Activated tumor necrosis factor receptor-associated factor molecules interact with IκB kinase releasing NF-κB family members from their cystolic inhibitors to enter the nucleus and initiate transcription of target genes.

In line with the phenotype of XLHED patients, EDAR pathway activation has primarily been linked to the window when appendages develop in utero. In mice, *Edar* mRNA is expressed from E14 in the developing epidermal basal layer, localized to preappendage placodes (Headon and Overbeek, 1999). The resultant EDAR protein remains localized to the placode into the final postnatal stages of HF development. In contrast, few studies have explored potential roles for EDAR signaling in adult tissue. Kowalczyk-Quintas et al. (2015) recently showed that *Edar* is expressed within the sebaceous glands of adult mice, and Inamatsu et al. (2006) reported *Edar* expression in the epidermal cells surrounding the dermal papilla. Moreover, Fessing et al. (2006) described EDAR expression in the secondary hair germ of telogen HFs, proposing that EDAR signaling is important for adult hair cycle (HC) regulation, particularly control of catagen onset through the up-regulation of X-linked inhibitor of apoptosis.

Hair cycling and wound healing are both examples of when major morphogenic changes occur in adult skin, a tissue that is normally under strict homeostatic control. To achieve this, numerous “developmental” signaling pathways are “reused” in the adult tissue (Stenn and Paus, 2001). Recently, we demonstrated a novel link between HC and the speed of adult skin healing, with a near doubling of healing efficiency in skin containing anagen HC stage follicles (Ansell et al., 2011). This led us to hypothesize an as yet unidentified role for the EDAR signaling pathway in adult skin wound healing. This hypothesis is supported by a case study from Barnett et al. (1979) describing poor skin graft healing in an XLHED patient.

Here we provide functional demonstration that EDAR signaling plays an important role in adult skin wound healing. Specifically, mice lacking the ligand EDA (*Tabby*) displayed reduced healing ability, whereas EDAR signaling augmentation promoted healing, not only in *Tabby* but also in wild-type (WT) mice. EDAR signaling manipulation altered multiple aspects of healing, including peri-wound proliferation, epidermal migration, and collagen deposition. Finally, we show that EDAR stimulation is able to promote human skin healing and is thus an attractive target for future therapeutic manipulation.

## Results

**Figure 1:**
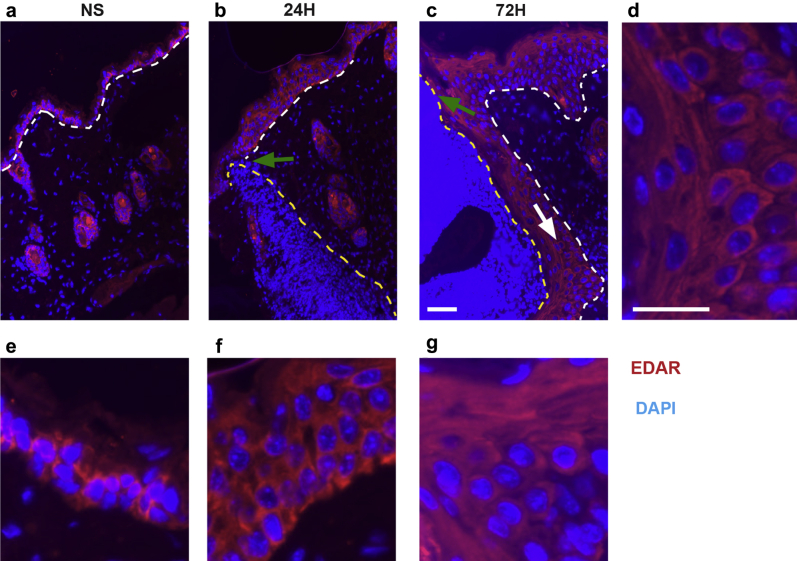
**Edar expression increases at the wound edge.** (**a**) In normal (unwounded skin), Edar immunoreactivity can be observed in the basal layer of the epidermis and within the hair follicles. (**b**) In a 24-hour wound, immunoreactivity appears to be present in basal and suprabasal layers of the peri-wound interfollicular epidermis. (**c**) In a 72-hour wound, immunoreactivity is apparent in peri-wound interfollicular epidermis. Staining intensity appears elevated in keratinocytes within the neo-epidermis, which appear to be actively migrating beneath the scab (white arrow and **d**). White dotted line denotes basement membrane separating interfollicular epidermis and dermis. Yellow dotted line indicates wound tissue or scab. Green arrow denotes wound edge. (**e–g**) Enlargement of images shown in **a–c**. Bar = 50 μm (**a–c**) and 20 μm (**d–g**). EDAR, ectodysplasin A receptor.

**Figure 2:**
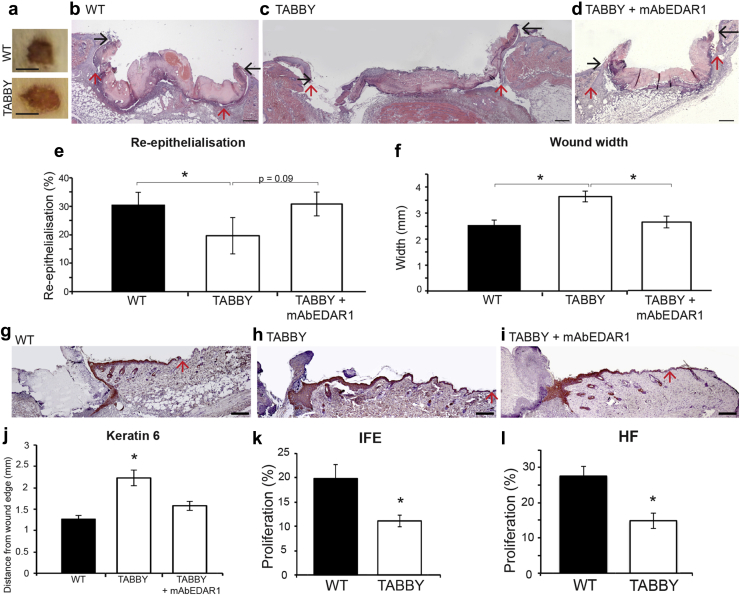
**Absence of EDAR signaling delays murine wound healing, which can be rescued by acute restoration of EDAR signaling.** (**a–d**) Representative macroscopic (**a**) and microscopic (**b–d**) hematoxylin and eosin–stained images of 6-mm excisional wounds at day 3 after wounding from wild-type (WT), Tabby, and mAbEDAR1-treated Tabby mice. All experimental groups were in an equivalent telogen stage of the hair cycle. Black arrows denote wound margin. Red arrows denote the neo-epidermal front. (**e, f**) Quantification of histological wound parameters reveals significantly delayed healing in Tabby mice, which can be rescued to WT levels by administration of mAbEDAR1. (**g–i**) Hyperproliferation at the wound edge, (marked by keratin 6 immunohistochemistry) is extended in Tabby mice (**h**) compared to WT (**g**), but can be restored by administration of mAbEDAR1 (**i**) and quantified (**j**). (**k, l**) Proliferation is significantly less in Tabby peri-wound interfollicular epidermis (**k**) and HF (**l**) than WT, as quantified by BrdU incorporation assay. Values are given as mean ± standard error of the mean. n = 6. EDAR, ectodysplasin A receptor; IFE, interfollicular epidermis; HF, hair follicle. *P < 0.05. Bar = 200 μm.

*Eda* null (*Tabby*) mice exhibit delayed wound healing, which can be restored by acute pathway activation. First, we proposed that a role for Edar signaling during wound healing would likely be reflected in wound edge induction. Thus, we analyzed Edar expression by immunofluorescence in both unwounded and wounded skin (Figure 1). We noted immunoreactivity with an anti-Edar antibody in the epidermis of unwounded skin (Figure 1a and e), which appeared expanded in the peri-wound interfollicular epidermis (IFE) of 24- (Figure 1b and f) and 72- (Figure 1c–g) hour wounds. To test the hypothesis that EDAR signaling was necessary for timely healing, we first examined the rate of wound repair in *Eda* null (*Tabby*) mice. We report significantly delayed excisional wound healing in the absence of EDA (Figure 2). *Tabby* wounds were larger than those in WT both macroscopically (Figure 2a) and microscopically (Figure 2b and c), quantified by an increased wound width and delayed rate of re-epithelialization (Figure 2e and f). To confirm that this healing delay was due to EDAR signaling deficiency and not phenotypic differences in *Tabby* skin, we also performed in utero correction of the *Tabby* phenotype using the validated EDAR-activating antibody mAbEDAR1 (Gaide and Schneider, 2003, Kowalczyk et al., 2011). Healing in adult mAbEDAR1-rescued *Tabby* mice (i.e., phenotypically normal but EDA deficient) remained delayed and indistinguishable from nonrescued *Tabby* mice (data not shown). Thus, developmentally specified structural changes in *Tabby* skin are unlikely to contribute to the observed adult wound healing phenotype.

Finally, we explored the effect of locally activating Edar signaling in adult *Tabby* mouse wounds. Here, mAbEDAR1 administered directly to the wound site (by subcutaneous injection) 24 hours before injury entirely rescued the healing delay in *Tabby* mice (Figure 2d–f). Specifically, the rate of re-epithelialization is increased, and wound width is significantly decreased compared to *Tabby*, generating a healing phenotype more in line with WT wounds. In line with previous delayed healing murine models, including the HF-deficient tail model (Langton et al., 2008), we observed extended epidermal activation (keratin 6 expression) in *Tabby* wounds (Figure 2j) (Langton et al., 2008). Local administration of mAbEDAR1 fully rescued this phenotype, restoring normal peri-wound IFE expression of keratin 6 (Figure 2g–j). Induction of wound edge epithelial proliferation is a key aspect of HC-modulated healing (Ansell et al., 2011). Peri-wound epithelial proliferation, measured by BrdU incorporation assay, was significantly decreased in *Tabby* mice compared to WT in both IFE and HF (Figure 2k and l).

**Figure 3:**
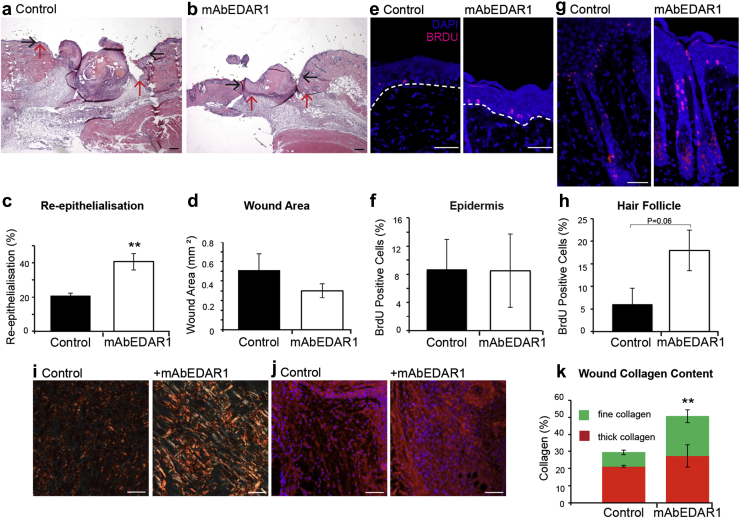
**EDAR signaling activation promotes healing in wild-type mice.** (**a, b**) Representative histology of C57 Bl/6 mouse wounds collected 3 days after wounding locally treated with mAbEDAR1 or vehicle (phosphate buffered saline) at the time of wounding. Black arrows denote wound edge. Red arrows denote migration of neo-epidermal tongue. (**c, d**) Quantification of healing parameters reveals faster healing following mAbEDAR1 treatment. (**e, f**) BrdU incorporation assay indicates that mAbEDAR1 treatment does not alter proliferation within the peri-wound interfollicular epidermis (marked by white dashed line). (**g, h**) Instead a strong trend toward increased proliferation was observed in peri-wound hair follicles. (**i**) Quantification of picro Sirius red staining (**k**) to reveals significantly higher granulation tissue collagen content in mAbEDAR1-treated mice with a specific increase in fine collagen fibers as shown by green or yellow staining. (**i, j**) Representative immunofluorescence for collagen 3 mirrors data shown in **k**. Values are given as mean ± standard error of the mean. n = 5. EDAR, ectodysplasin A receptor. **P < 0.01. Bar = 150 μm (**a, b**), 25 μm (**e**), 30 μm (**g**), 100 μm (**i**), 50 μm (**j**).

Activation of EDAR signaling accelerates healing in WT mice. To further explore the therapeutic potential of EDAR signaling activation, we next administered mAbEDAR1 locally to the wound site 24 hours before wounding in WT mice. WT mAbEDAR1-treated wounds displayed accelerated healing, with a clear induction of re-epithelialization (Figure 3a–d). Peri-wound proliferation levels were assessed by BrdU incorporation assay. In contrast to *Tabby* mice, mAbEDAR1-treated WT mice displayed increased proliferation only in peri-wound HFs and not IFE (Figure 3e–h). During development, EDA/EDAR is essential for epidermal/dermal cross-talk required for effective appendage morphogenesis. Thus, EDAR signaling could have nonepidermal roles in skin homeostasis and repair. Indeed, careful analysis revealed increased wound collagen content in mAbEDAR1- versus placebo-treated WT mice (Figure 3i and k). Specifically, picro Sirius red staining revealed increased collagen deposition in mAbEDAR1-treated wounds, in addition to a defined increase in the proportion of fine (green) fibers (Figure 3i and k). Immunofluorescence for Col3a1 confirms increased wound bed collagen in mAbEAR1-treated mice, despite no reports of active EDAR signaling in fibroblasts (Figure 3j). We note that in a previous transcriptional study, Cui et al. (2002) reported up-regulation of Col1a1 and Col3a1 in adult *Tabby* skin. However, they also reported increased expression of Col3a1 in EDA overexpressing (EDA-A1) transgenic mice, suggesting complex paracrine signaling (Cui et al., 2002).

**Figure 4:**
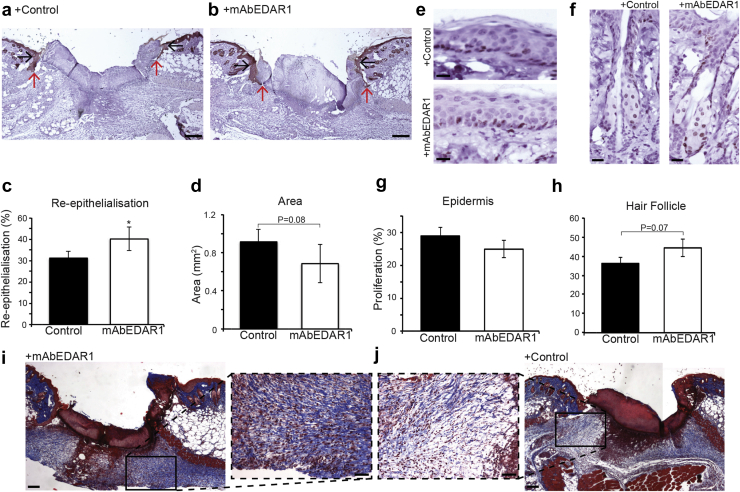
**EDAR signaling activation promotes healing in ovariectomized (OVX) mice.** (**a, b**) Representative histology of C57 Bl/6 OVX mouse wounds collected 3 days after wounding locally treated with mAbEDAR1 or vehicle (phosphate buffered saline) at the time of wounding. Black arrows denote wound edge. Red arrows denote migration of neo-epidermal tongue. (**c, d**) Quantification of healing parameters reveals faster healing following mAbEDAR1 treatment. (**e, f**) Representative Ki67 immunohistochemistry revealing no difference in interfollicular epidermal proliferation but increased HF proliferation in mAbEDAR1-treated OVX mice, quantified in **g–j**. Masson trichrome staining reveals increased collagen deposition in mAbEDAR1-treated OVX wounds compared to control treated. Insets show magnified collagen fibers (blue). Values are given as mean ± standard error of the mean. n = 5. EDAR, ectodysplasin A receptor. *P < 0.05. Bar = 200 μm (**a, b**), 150 μm (**e, f**), 200 μm (**i, j**), 50 μm (insets).

EDAR signaling activation restores healing in ovariectomized (OVX) mice. OVX mouse provides a widely used, physiologically relevant model of delayed healing (Emmerson and Hardman, 2012, Hardman et al., 2008) in which to test the beneficial effects of EDAR signaling activation. Here, OVX mice received mAbEDAR1 or placebo local to the wound site 24 hours before wounding. In comparison to control, OVX mice treated with mAbEDAR1 displayed significantly accelerated healing, with increased re-epithelialization and decreased wound area compared to control (Figure 4a–d). Similarly to WT mice we observed no differences in peri-wound IFE proliferation but a strong trend toward increased peri-wound HF proliferation (Figure 4e–h) in mAbEDAR1-treated OVX mice. This intriguing HF-specific peri-wound proliferative response is in line with the findings of Fessing et al. (2006), who reported a role for EDAR signaling in adult HF cycling.

Given that both epidermal and dermal aspects of healing were affected in WT mice, we investigated the collagen content of OVX wounds by Masson trichrome staining. Here, collagen content in OVX +mAbEDAR1 is greater than in placebo-treated mice (Figure 4i and j).

**Figure 5:**
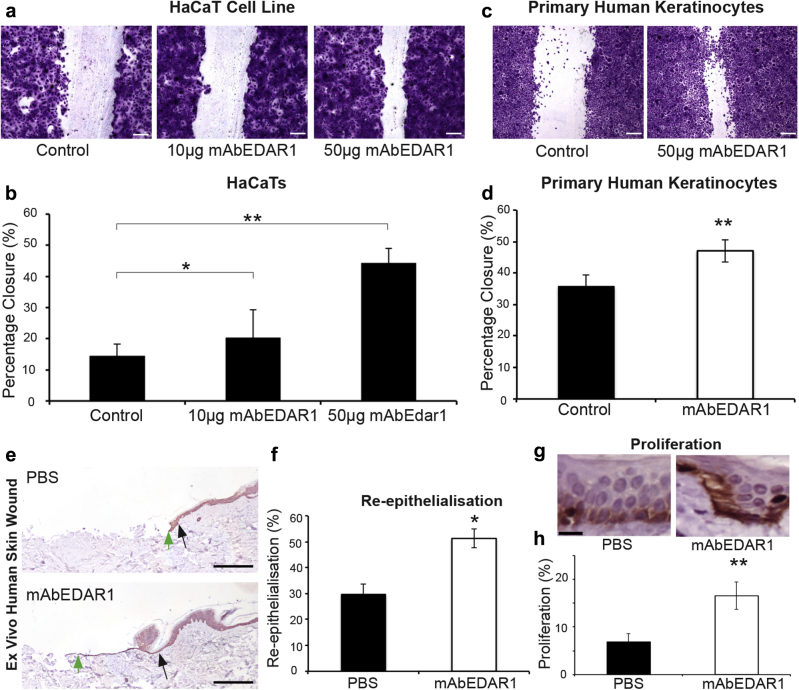
**EDAR signaling promotes human wound healing.** (**a, b**) In vitro treatment with mAbEDAR1 (10 μg or 50 μg in 500 μl of media) promotes human keratinocyte cell line (HaCaT cells) scratch wound closure (24 hours after scratch) compared to phosphate buffered saline (PBS)–treated cells. (**c, d**) In vitro treatment with mAbEDAR1 (50 μg in 500 μl of media) promotes primary human keratinocyte scratch closure compared to Aprily-treated control cells. (**e, f**) The 3-mm partial-thickness biopsy wounds in ex vivo cultured skin were treated with mAbEDAR1 (100 μg in 10 μl of PBS) or PBS and left to heal ex vivo. (**e**) Images display representative keratin 14 staining. Black arrows denote wound edge. Green arrows denote furthest point of neo-epidermal tongue. (**f**) Quantification of accelerated healing at 3 days after wounding. (**g, h**) Proliferation is increased in human ex vivo skin wounds treated with mAbEDAR1 compared to PBS-treated wounds, quantified from Ki67 immunohistochemistry (**g**). Values are given as mean ± standard error of the mean. n = 4. EDAR, ectodysplasin A receptor. *P < 0.05; **P < 0.01. Bar = 200 μm (**a, c, e**), 20 μm (**g**).

Activation of EDAR signaling promotes human healing. The EDAR signaling pathway is highly conserved between mouse and human, as highlighted by the similarities in the XLHED and *Tabby* developmental phenotypes. However, no study to date has demonstrated a role for EDAR signaling in adult human tissue. We thus assessed the potential of EDAR activation to promote human skin wound healing. First, mAbEDAR1 treatment in the EDAR-responsive human HaCaT keratinocyte cell line (Swee et al., 2009) resulted in a statistically significant dose-dependent increase in scratch wound closure, demonstrating that human keratinocytes are competent to respond to exogenous pathway activation (Figure 5a and b). Increased scratch wound closure may indicate that activation of Edar signaling can increase cell proliferation in vitro. Although we did not find this to be the case, careful analysis of cell migration in vitro indicates that Edar signaling can increase cell motility (see Supplementary Figure S1 online). We also investigated the ability of primary human keratinocytes to respond to mAbEDAR1 treatment and found that scratch wound closure in this model was also significantly accelerated (Figure 5c and d). To confirm efficacy at the physiological level, we turned to the validated human whole skin ex vivo wound model (Stojadinovic and Tomic-Canic, 2013). In this ex vivo partial-thickness human skin wound model, direct topical administration of mAbEDAR1 significantly increased both wound re-epithelialization (Figure 5e and f) and peri-wound proliferation (Figure 5g and h).

## Discussion

Timely wound healing requires the communication of multiple cell types, a phenomenon for which the EDAR pathway is essential during in utero development, as highlighted by the XLHED disease phenotype. This study discusses a previously unreported direct link between EDAR signaling and wound healing. We found immunoreactivity with an anti-Edar antibody to be increased in peri-wound IFE compared to unwounded IFE, and, more importantly, in the absence of active EDAR signaling wound healing was significantly delayed. Moreover, local activation of EDAR signaling promoted healing in EDAR-deficient mice, a non–EDAR-linked mouse model of delayed healing and crucially in ex vivo human wounds. Surprisingly, EDAR deficiency/treatment effects are not exclusively confined to the epidermis but also manifest in the dermis.

Reports of EDAR expression in adult murine skin (Fessing et al., 2006, Kowalczyk-Quintas et al., 2015) suggested an active role for EDA/EDAR signaling postnatally. Given that the role of EDA/EDAR signaling is predominantly in the rearrangement of the epidermis into placodes during development (Ahtiainen et al., 2014, Mustonen et al., 2004, Schmidt-Ullrich et al., 2006), we focused on epithelial aspects of the wound phenotype, reporting EDAR-dependent changes in both peri-wound proliferation and re-epithelialization. The re-epithelialization phenotype particularly fits with the report of Mustonen et al. (2004) in which overactivation of EDAR (K14tg-EDAR mice) during development resulted in an increase in placode size, that is, an EDAR-dependent increase in cell migration. Our in vitro analysis of cell motility provides further evidence that Edar signaling may influence cell migration during re-epithelialization.

Our analysis of proliferation in mAbEDAR1-treated WT mice revealed no difference in wound edge IFE proliferation but an clear EDAR dependency of wound edge HF proliferation. That HF cells would be more capable of responding to EDAR signaling is in line with the findings of Fessing et al. (2006), who described a postnatal role for EDAR in HC. We note with interest that during development, pre-appendage placode proliferation was unaltered in K14-EDAtg mice (Mustonen et al., 2004). Thus, it seems that the EDAR pathway has greater influence over appendage proliferation in adulthood than development, or that additional wound-derived signals contribute to the observed effects in adult skin. HF cell involvement in wound healing has been well characterized in several elegant studies (Ito et al., 2005, Levy et al., 2007, Mardaryev et al., 2011, Page et al., 2013), and it is possible that Edar signaling may regulate the involvement of different HF cell populations. This is particularly interesting in the context of HF stem cell contribution to healing, which seems o be influenced by the stage of repair.

Intriguingly, we also observed increased peri-wound sebaceous gland proliferative response in mAbEDAR1 treated mice (data not shown). Mechanistically, the proliferation inducing effects of EDAR are most likely explained by the pathway convergence on Cyclin D1, which will drive cell cycle progression (Schmidt-Ullrich et al., 2006). More interesting still, it remains unclear what signals are involved in communicating an injury response signal to peri-wound follicles. While several candidates have been suggested (Chen et al., 2015) it is tempting to speculate that EDAR may be involved in this initial wound-HF cross-talk. Here, we note with interest that Inamatsu et al. (2006) have previously reported Edar-expressing ectopic HFs when fetal dermis is recombined with adult epidermis, further supporting the concept that adult skin can reactivate embryonic processes under specific circumstances (Inamatsu et al., 2006).

That we observe alterations in dermal aspects of healing when the EDAR pathway is stimulated was unexpected, given that there are no reports of EDAR expression in fibroblasts. Increased wound collagen following mAbEDAR1 treatment could be explained by secondary signaling and epidermal-dermal cross-talk. A more simple explanation would be that this could be a reflection of a more progressed stage of healing. However, observations of Cui et al. (2002), who report an increase in collagen mRNA expression in EDA-overexpressing mice, suggest the former explanation.

The highly conserved nature of the EDAR pathway (Iida et al., 2014, Lough et al., 2013, Pantalacci et al., 2008) led us to test whether the effects of EDAR signaling on wound healing would also be applicable to human skin healing. EDAR activation in the EDAR-expressing (Swee et al., 2009) HaCaT cell line (human keratinocyte) increased the rate of scratch wound closure. Moreover, in primary human keratinocytes and the whole human skin ex vivo wound model, we also observed a marked improvement in re-epithelialization after EDAR signaling activation. To our knowledge this is a previously unreported demonstration that EDAR signaling can directly influence keratinocyte proliferation and migration within human skin.

In summary, our data reveal a previously unidentified role for EDA/EDAR signaling in adult cutaneous wound healing in both mice and humans. Surprisingly we show the EDA/EDAR pathway to be involved in multiple aspects of healing, despite EDAR expression being epidermally restricted. However, the complex role of this pathway in coordinating HF morphogenesis, in which both epidermal and dermal skin components contribute, fully support our observations (Mustonen et al., 2004, Schmidt-Ullrich et al., 2006). In a prior publication we demonstrated a strong link between HC stage and wound healing outcome (Ansell et al., 2011). The concept of manipulating HC to promote wound healing is experimentally attractive but likely to prove clinically challenging (Jimenez et al., 2012, Jimenez et al., 2015). We suggest that targeting the EDA/EDAR pathway offers a more practical, therapeutically attractive solution.

## Materials and Methods

### Wounding

All animal procedures were approved by the UK Home Office (Project License 40/3202) after local ethical approval. Seven-week-old male *Tabby* (FVB/N) or WT mice (FVB/N or C57) were anaesthetized (isoflurane) and wounded following our established protocol (either two dorsal full-thickness 1-cm incisions or two full-thickness 6-mm excisions) (Ansell et al., 2014). Mice were individually housed postoperatively and left to heal by secondary intention with analgesia (buprenorphine). Anti-EDAR1 monoclonal mouse IgG1 (mAbEDAR1) or placebo (Aprily1 isotype-matched antibody or phosphate buffered saline) was administered 24 hours before wounding (2 mg/kg) via subcutaneous injection at the wound site. BrdU was administered intraperitoneally 2 hours before sacrifice. Three days after wounding, samples (wounds and skin) were excised, bisected, and fixed in 10% buffered formalin (histology) or snap frozen (biochemical analysis).

### Tissue processing, histology, and analysis

Histological sections were prepared from fixed, paraffin-embedded tissue. Five-micrometer sections were stained with hematoxylin and eosin (Vector, Peterborough, UK), Sirius red (in picric acid), or Masson trichrome, or underwent immunohistochemical analysis with the following antibodies: anti-Edar (R&D, Abingdon, UK, Systems AF745) anti-keratin 6 (Covance, NJ, PRB-169P), anti-keratin 14 (Covance PRB-155P-100), anti-BrdU (Abcam, ab6326), or anti-Col3a1 (Santa Cruz, Heidelberg, Germany, sc28888). Primary antibody incubation was followed by the appropriate biotinylated antibody, ABC reagent and NovaRed (Vector) mounted in Pertex (CellPath, Newtown, UK) or streptavidin Cy3 secondary (Sigma-Aldrich, Poole, UK) mounted in Mowiol containing DAPI (Sigma-Aldrich). Images were captured as follows: bright field—Eclipse E600 microscope (Nikon, Kingston-upon-Thames, UK) and spot camera (Image Solutions, Preston, UK); picro Sirius red—plane polarized light microscope/camera (Leica, Milton Keynes, UK); fluorescence—Leica MDLB/camera (Coolsnap, Photometrics, Tucson, AZ). Image analysis was performed using ImagePro Plus (Media Cybernetics, Rockville, MD) or Metamorph software and Corel Paintshop Pro (Ottowa, Canada). Wound measurements were made from hematoxylin and eosin-stained images. Percentage re-epithelialization was quantified as follows: (distance traveled by migrating epithelial tongue/total distance to travel)*100. Wound width was quantified as the distance between normal dermal architecture at the edge of each wound, and wound area was quantified as the area of granulation tissue beneath the scab.

### In vitro scratch wounding

The HaCaT human keratinocyte cell line was cultured at 37 °C, 5% CO_2_ in DMEM (Sigma-Aldrich) with 10% fetal bovine serum (Sigma-Aldrich). Scratch wounds were generated in a confluent cell layer using a 1-ml sterile pipette tip. mAbEDAR1 (10 μg, 50 μg) or phosphate buffered saline was added, and 24 hours later cells stained with crystal violet. Images were captured (Eclipse E600 microscope; Nikon) and cell migration quantified (ImagePro Plus; Media Cybernetics). Cell migration was quantified as a percentage increase in closure compared to scratches photographed at 0 hours, and measurements were taken at 30 individual points across a single scratch and averaged. Primary human keratinocytes were isolated from adult female abdominal skin, cultured and scratched as described earlier.

### Ex vivo human skin wounding

Ex vivo methodology was as previously described (Stojandinovic and Tomic-Canic, 2013) using tissue collected after ethical approval was received. Briefly, adult female abdominal skin was washed in sterile phosphate buffered saline (Sigma-Aldrich) and excess fat removed. Each 8-mm-diameter biopsy-punched construct was partial thickness wounded with a 3-mm punch. Constructs were then cultured at the air-liquid interface in 1% antibiotic-antimycotic/10% fetal bovine serum-supplemented DMEM (Sigma-Aldrich). mAbEDAR1 or Aprily1 was applied directly to the central punch wound. Biopsies were maintained at 37 °C, 5% CO_2_ for 3 days, then formalin fixed before tissue processing and sectioning as described earlier.

### Analysis of cell motility

#### Plate preparation

HaCaT cells at P41 were seeded into wells of a 24-well plate at 1.5 × 10^4^ cells per well. Just before imaging, cells were treated with either mAbEdar1 or Aprily and imaged for 24 hours under wide-field live imaging microscope. Cell migration was then analyzed as either track displacement (distance between start point and endpoint) or track length (total distance traveled by the cell) using a Wavelet plugin on Imaris (Egor Zindy, Zurich, Switzerland).

### Statistical analysis

Statistical differences were determined by the Student *t* test (SPSS). *P* < 0.05 was considered significant.

### Study approval

All animal work was approved by the UK Home Office (Project License 40/3202) following local ethical approval. Human skin donors provided written consent before undergoing surgery, and tissue was handled and stored in accordance with the Human Tissue Act (2004).

## Conflict of Interest

PS and NK are shareholders of Edimer Pharmaceuticals. KMH is employed by Edimer Pharmaceuticals. NK is a director and employee of Edimer Pharmaceuticals.
