# Supplementary material for: Ectodysplasin A Pathway Contributes to Human and Murine Skin Repair
Source: J Invest Dermatol. 2016 May;136(5):1022–30. doi: 10.1016/j.jid.2015.09.002 (PMC4967474; doi:10.1016/j.jid.2015.09.002)
Supplement: Supplementary Figure S1 [file mmc1.pdf]

S1

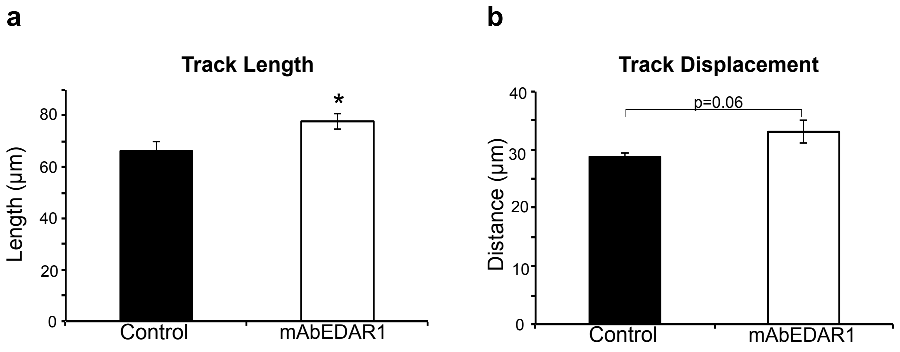

Figure S1 Activation of Edar signalling increases cell motility.
a, Total distance travelled over a period of 24 hours is significantly greater in mAbEDAR1 treated HaCaTs than control treated. b, Trend-towards increased directional cell motility upon activation of Edar signalling. Track displacement denotes distance between start and end position. Bar shows mean ± SEM, n=3, *P<0.05.
